# Direct Generation of Electric Currents from Flowing Neutral Ionic Solutions

**DOI:** 10.1155/2013/320427

**Published:** 2013-09-25

**Authors:** Boyang Wang

**Affiliations:** Institute of Chemistry, and Institute of Theoretical Physics, Chinese Academy of Sciences, No. 2 North Zhongguancun 1st Road, Beijing 100190, China

## Abstract

We have discovered a new method of generating electric currents, directly from high pressure-induced flow of neutral ionic solutions. The mechanism is that the cations and anions have different flow velocities, if their atomic masses are dramatically different, due to different accelerations generated from the high applied pressure. The generated electric current is very sensitive to the strengths of the applied pressure, and it might be potentially used for detection of atomic masses and pressures.

Production and storage of electricity are among the most important activities in modern industrial processes. Conventional methods of generating electric current are electromagnetic induction, photovoltaic [1], thermoelectric [2], piezoelectric [3], electrokinetic generation [4], and so on. Recently, nanoscale electric devices have been developed extensively, such as nanoscale thermoelectric [5–7] and piezoelectric [8–10] devices. Nanoscale electrokinetic generation of electric current has also had a great progress, both in theories [11–13] and experiments [14–17]. Other methods of generating electric currents at the nanoscale have also been proposed, such as using electron tunnelling phenomena [18]. These methods of current generation usually require usage of solid materials with special electric response to external light, thermal and mechanical excitations, or require applying strong external electric fields. Therefore, the mechanisms of current generation often involve multisteps of processes, and the practical instruments for these methods might be also complicated.

In this work, we have discovered that electric currents can be directly generated from fast-flowing neutral ionic solutions under external pressures, using molecular dynamics (MD) simulations. The current is the result of dramatically different atomic masses, and hence very different accelerations, of the counterions in the flowing ionic solutions. This method represents a new type of one-step electric current generation, with possible applications in convenient electric energy storage, and highly sensitive detection of very large pressures.

In our MD simulations using the NAMD software package [19] and the CHARMM27 force field [20], a pair of counterions and 7948 water molecules were initially positioned inside a 6.2 × 6.2 × 6.2 nm simulation box, with periodic boundary conditions applied, as shown in Figure 1. The water molecules were simulated using the TIP3P model [21]. The cation was N-heptyl-pyridine, and the anion was chloride. The partial charges of the atoms in the cation were calculated by ab initio methods, and the force field parameters were estimated similar to those in [12] Pressure-induced flows of the ionic solution were generated by applying an external force of 0.02 kcal/(mol·Å) to all the nonhydrogen atoms in the region of −0.5 nm < *x* < 0.5 nm, in the positive *x* direction. Given the number of water molecules and ions in the simulation box, this generates a pressure of *P* ≈ 500 atm [11]. Langevin dynamics were performed, with 0.1 ps^−1^ damping coefficient, which might be correct for polar groups and water, at *T* = 300 K [7]. The induced flow velocity of water molecules reached a constant value of around 85 m/s. When the atomic mass of the anion, *M*, was much larger than the atomic mass of any atom in the cation, the anion's flow velocity became smaller than the cation, because of its smaller acceleration, supposing that the anion and cation were under the same external pressure, and the surface areas of the nonhydrogen atoms in the cation and the anion were not very different. Therefore, as confirmed by our simulations, this method of current generation mainly depends on the atomic masses of the counterions, but does not qualitatively depend on the chemical structures or signs of charges of the counterions. This method could be applied similarly to the cases when the counterions are very large, such as colloids, but those are not the focus of this work.

We have estimated the influence of the anion's atomic mass *M* on its flow velocity. In the simulations, we only changed the atomic mass of the anion from 35.5 Dalton, to 62.5, 125, 200, and 250 Dalton and left all the other parameters unchanged. The velocities of the cation were very close to the average velocities of the water molecules, since the atomic masses of the atoms in the cation were close to those of the atoms in water molecules. In Figure 2(a), we plot the flow velocities of the cation and the anion, depending on different values of *M*. We can see that in general, the cation flowed faster than the anion, and the current was noticeable. The current first decreased and later increased with *M*. The results were obtained from the simulations of pressurizing the solution for *t* ≈ 20 ns, during which the ions pass the periodic boundary by *≈*300 times. The flow velocities of the water molecules and the counterions remained almost constant during the flow of the solutions. In Figure 3, we plot the distance travelled by the chloride anion *L*, when *M* = 35.5 Dalton, depending on the length of the simulation. From the figure, we can see that the flow velocity of the chloride ion remained almost constant within about 4 ns of simulation. Please note that at the nanoscale, small molecules are subject to fluid pressure similar to single atoms. Although there are chemical bonds between atoms in a molecule, the fluid pressure will be distributed everywhere, including the internal region of molecules, so the atomic mass has the major influence on the flow velocity of ions, and chemical structures of ions are less important.

To study the generation of electric current in a nanoscale confinement, we also simulated the flow of the ionic solution with the same counterions and 6659 water molecules, inside a 6.2 nm long (76,0) carbon nanotube (diameter of 6.03 nm). The system was also positioned inside a 6.2 × 6.2 × 6.2 nm simulation box, with periodic boundary conditions applied. We also only changed the values of *M* and plotted the velocities of the cation and the anion depending on the values of *M* in Figure 2(b). We can see that the cation also flowed faster than the anion, and the current first increased and later decreased with *M*. We only simulated the condition when the N-heptyl-pyridine cation was always totally solvated and surrounded by water molecules, away from the carbon nanotube surface. Because the cation has a hexagonal ring and a hydrophobic tail, it might attach to the hydrophobic surface of the carbon nanotube from time to time. In this case, the cation would have frictions with the nanotube surface. But the cation might prefer to be totally surrounded by water molecules, due to the entropic effects, if the concentration of the ionic solution is low enough. 

We also simulated the pressure-induced flows of the same ionic solutions, both as bulk and inside the carbon nanotube, at a much larger pressure of *P* ≈ 2000 atm, to see the effects of the pressure on the induced electric currents. The velocities of the cation and the anion are plotted in Figure 4, depending on the values of *M*. In Figure 4(a), we can see that in bulk solutions, the cation flowed faster than the anion in the bulk solution, and the electric current always increased with the value of *M*. The electric currents were nearly 3 times larger than the currents at *P* ≈ 500 atm, when *M* ≥ 125 Dalton, because a larger pressure caused a larger difference in the accelerations, and thus flow velocities, of the cation and the anion. In Figure 4(b), we can see that for the ionic solutions confined in the carbon nanotube, the cation flowed faster than the anion in the bulk solution, and the current first decreases and later increases with the value of *M*. The electric currents were approximately 5 to 10 times larger than the currents at *P* ≈ 500 atm, when *M* ≥ 200 Dalton. The above results show that the generated electric current was very sensitive to the atomic mass of the anion, as well as the strength of the applied pressure, and the current could be a powerful tool to detect the atomic masses of the ions in the solutions, as well as the external applied pressures. This method of detection inside ionic solutions could be highly focused in a nanoscale region. This method is also superior in that it is a one-step sensing of atomic masses and external pressures and does not involve any unnecessary mechanisms of intermediate transformation of signals, which might induce system errors in the detections. The induced electric current might also be very sensitive to the system's temperature, and we plan to investigate this in the future.

In summary, we have discovered a method to directly generate electric currents from fast-flowing neutral ionic solutions. This method is very convenient and has a simple mechanism. It could have potential applications in power generation and storage, as well as highly sensitive nanoscale detection of pressures and atomic masses [22–26]. It also provides a new possibility for separating mixed heavy element nuclear materials into different pure heavy elements.

## Figures and Tables

**Figure 1:**
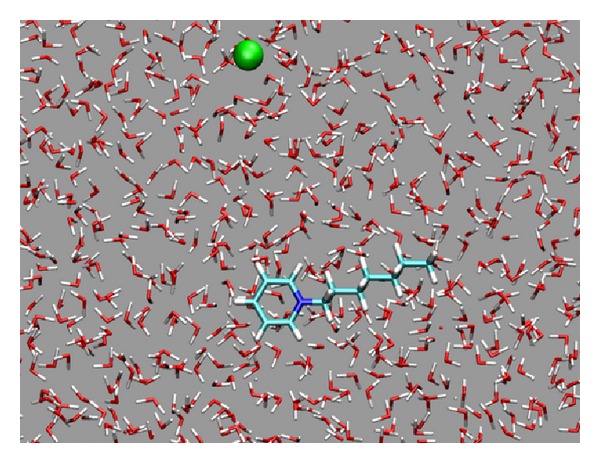
Initial configuration of the simulated system. Two counterions were located randomly in the water box. The cation was nonprotonated N-heptyl-pyridine, and the anion was chloride ion (in green).

**Figure 2:**
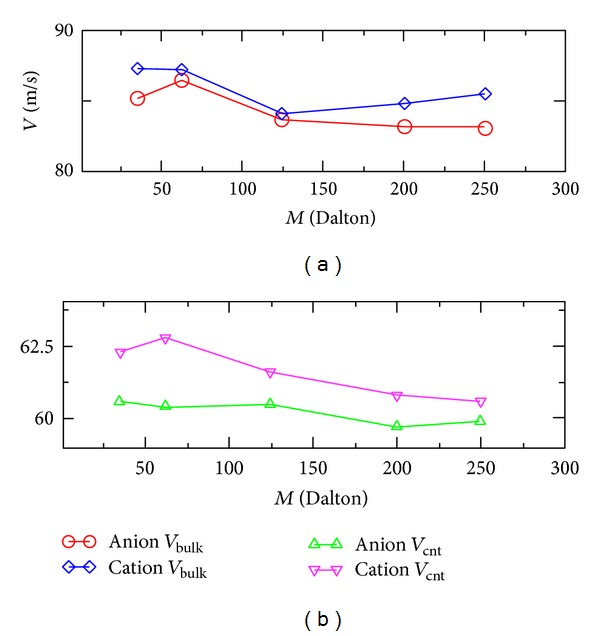
The flow velocities of the cation and anion depending on the different atomic masses of the anion, at an external pressure of *P* ≈ 500 atm and at temperature *T* = 300 K. (a) Flow velocities of the cation and the anion in bulk solution. (b) Flow velocities of the cation and the anion in a (76,0) carbon nanotube.

**Figure 3:**
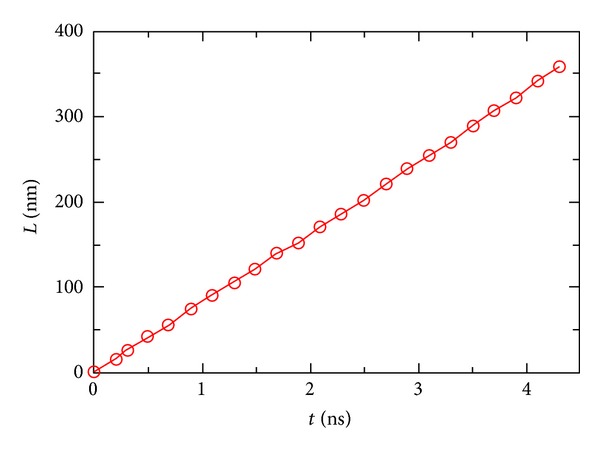
The distance *L* travelled by the chloride ion (*M* = 35.5 Dalton), depending on the length of the simulation *t*, at an external pressure of *P* ≈ 500 atm and at temperature of *T* = 300 K.

**Figure 4:**
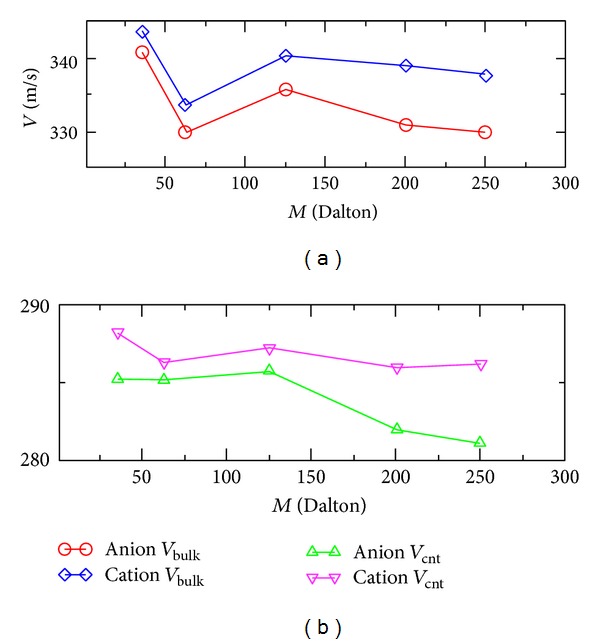
The flow velocities of the cation and anion depending on the different atomic masses of the anion, at a pressure of *P* ≈ 2000 atm and at temperature of *T* = 300 K. (a) Flow velocities of the cation and the anion in bulk solution. (b) Flow velocities of the cation and the anion in a (76,0) carbon nanotube.

## References

[1] Jacobson MZ (2009). Review of solutions to global warming, air pollution, and energy security. *Energy and Environmental Science*.

[2] Rowe DM (2006). *Thermoelectrics Handbook: Macro to Nano*.

[3] Gautschi G (2002). *Piezoelectric Sensorics: Force, Strain, Pressure, Acceleration and Acoustic Emission Sensors, Materials and Amplifiers*.

[4] Dukhin SS, Derjaguin BV *Electrokinetic Phenomena*.

[5] Hone J, Ellwood I, Muno M (1998). Thermoelectric power of single-walled carbon nanotubes. *Physical Review Letters*.

[6] Boukai AI, Bunimovich Y, Tahir-Kheli J, Yu J-K, Goddard WA, Heath JR (2008). Silicon nanowires as efficient thermoelectric materials. *Nature*.

[7] Yu C, Kim YS, Kim D, Grunlan JC (2008). Thermoelectric behavior of segregated-network polymer nanocomposites. *Nano Letters*.

[8] Wang ZL, Song J (2006). Piezoelectric nanogenerators based on zinc oxide nanowire arrays. *Science*.

[9] Kong XY, Wang ZL (2003). Spontaneous polarization-induced nanohelixes, nanosprings, and nanorings of piezoelectric nanobelts. *Nano Letters*.

[10] Král P, Mele EJ, Tománek D (2000). Photogalvanic effects in heteropolar nanotubes. *Physical Review Letters*.

[11] Král P, Shapiro M Nanotube electron drag in flowing liquids. *Physical Review Letters*.

[12] Wang B, Král PJ (2006). Coulombic dragging of molecules on surfaces induced by separately flowing liquids. *Journal of the American Chemical Society*.

[13] Qiao R, Aluru NR (2005). Scaling of electrokinetic transport in nanometer channels. *Langmuir*.

[14] Ghosh S, Sood AK, Kumar N (2003). Carbon nanotube flow sensors. *Science*.

[15] Ghosh S, Sood AK, Ramaswamy S, Kumar N (2004). Flow-induced voltage and current generation in carbon nanotubes. *Physical Review B*.

[16] Liu Z, Zheng K, Hu L (2010). Surface-energy generator of single-walled carbon nanotubes and usage in a self-powered system. *Advanced Materials*.

[17] van der Heyden FHJ, Bonthuis DJ, Stein D, Meyer C, Dekker C (2006). Electrokinetic energy conversion efficiency in nanofluidic channels. *Nano Letters*.

[18] Wang B, Vuković L, Král P (2008). Nanoscale rotary motors driven by electron tunneling. *Physical Review Letters*.

[19] Phillips JC, Braun R, Wang W (2005). Scalable molecular dynamics with NAMD. *Journal of Computational Chemistry*.

[20] MacKerell AD, Bashford D, Bellott M (1998). All-atom empirical potential for molecular modeling and dynamics studies of proteins. *The Journal of Physical Chemistry B*.

[21] Jorgensen WL, Chandrasekhar J, Madura JD, Impey RW, Klein ML (1983). Comparison of simple potential functions for simulating liquid water. *The Journal of Chemical Physics*.

[22] Kong J, Franklin NR, Zhou C (2000). Nanotube molecular wires as chemical sensors. *Science*.

[23] Besteman K, Lee J-O, Wiertz FGM, Heering HA, Dekker C (2003). Enzyme-coated carbon nanotubes as single-molecule biosensors. *Nano Letters*.

[24] Modi A, Koratkar N, Lass E, Wei B, Ajayan PM (2003). Miniaturized gas ionization sensors using carbon nanotubes. *Nature*.

[25] Liu H, Kameoka J, Czaplewski DA, Craighead HG (2004). Polymeric nanowire chemical sensor. *Nano Letters*.

[26] Zheng G, Patolsky F, Cui Y, Wang WU, Lieber CM (2005). Multiplexed electrical detection of cancer markers with nanowire sensor arrays. *Nature Biotechnology*.

